# 

**Published:** 1874-08-01

**Authors:** 


					﻿No. XV.
CHICAGO, AUGUST 1, 1874.
Vol. XV.
THE
Medical Examiner.
A
Semi-Mont nN Journal of Medical Sciences.
TERMS.— Yearly Subscriptions, Three Dollars, in advance. Single Number Fifteen Cents. All Communi-
cations should be addressed to Publishers Medical Examiner, 65 Randolph st., cor. State, Chicago.
				

